# Olive Oil and the Hallmarks of Aging

**DOI:** 10.3390/molecules21020163

**Published:** 2016-01-29

**Authors:** Lucía Fernández del Río, Elena Gutiérrez-Casado, Alfonso Varela-López, José M. Villalba

**Affiliations:** 1 Department of Cell Biology, Physiology and Immunology, Agrifood Campus of International Excellence ceiA3, University of Córdoba, Campus Rabanales, Severo Ochoa Building, 14014 Córdoba, Spain; luciafernandezdelrio@gmail.com (L.F.R.); e.gutierrezcasado@gmail.com (E.G.-C.); 2 Department of Physiology, Institute of Nutrition and Food Technology “José Mataix”, Biomedical Research Center (CIBM), University of Granada, Avda. del Conocimiento s.n., Armilla, 18100 Granada, Spain; alvarela@ugr.es

**Keywords:** DNA damage, fatty acids, hallmarks of aging, Mediterranean diet, olive oil, oxidative stress, polyphenols, senescence

## Abstract

Aging is a multifactorial and tissue-specific process involving diverse alterations regarded as the “hallmarks of aging”, which include genomic instability, telomere attrition, epigenetic alterations, loss of proteostasis, deregulated nutrient sensing, mitochondrial dysfunction, cellular senescence, stem cell exhaustion and altered intracellular communication. Virtually all these hallmarks are targeted by dietary olive oil, particularly by virgin olive oil, since many of its beneficial effects can be accounted not only for the monounsaturated nature of its predominant fatty acid (oleic acid), but also for the bioactivity of its minor compounds, which can act on cells though both direct and indirect mechanisms due to their ability to modulate gene expression. Among the minor constituents of virgin olive oil, secoiridoids stand out for their capacity to modulate many pathways that are relevant for the aging process. Attenuation of aging-related alterations by olive oil or its minor compounds has been observed in cellular, animal and human models. How olive oil targets the hallmarks of aging could explain the improvement of health, reduced risk of aging-associated diseases, and increased longevity which have been associated with consumption of a typical Mediterranean diet containing this edible oil as the predominant fat source.

## 1. Introduction

Aging is a time-dependent progressive decline in physiological function of the organism that takes place with decreased fertility and increased susceptibility to endogenous and external threats, leading to a wide variety of related diseases such as degenerative and neoplastic disorders. Nowadays, it has been recognized that aging-related alterations are multifactorial and tissue-specific at the organismal level, and involve diverse processes regarded as the “hallmarks of aging”. These hallmarks include genomic instability, telomere attrition, epigenetic alterations, loss of proteostasis, deregulated nutrient sensing, mitochondrial dysfunction, cellular senescence, stem cell exhaustion and altered intracellular communication [1]. Scientists have demonstrated a strong interest not only in unravelling the causes of aging, but also in discovering how we can manipulate potential causes of aging to decrease, stop, or even revert its rate of progression [2,3]. Of note, a number of dietary approaches have revealed successful to decrease the incidence of aging-related diseases or to even increase lifespan.

Pro-survival properties of antioxidants have been shown in several disease models [4], although only a few interventions with antioxidants have successfully increased lifespan so far [5]. Calorie restriction without malnutrition is clearly the best characterized non-genetic intervention that increases maximum lifespan and improves healthspan, preventing or delaying the onset of pathophysiological changes in multiple species [6]. In addition, prospective studies have shown that adherence to a typical Mediterranean diet with olive oil (OO) as the predominant fat source is associated with improved health, lower mortality and increased longevity, reduced risk of cardiovascular diseases, cancer, and the incidence of age-related cognitive decline as Parkinson’s and Alzheimer’s disease [7]. These benefits have been attributed not only to its high monounsaturated fatty acid (MUFA) content but also to the healthy properties of minor though highly bioactive components, particularly in the case of virgin (VOO) and extra virgin olive oil (EVOO). These minor components can be classified into the unsaponifiable (non-polar) and the soluble (polar) fraction, which includes a variety of phenolic compounds such as hydroxytyrosol (HT), tyrosol, caffeic acid, oleuropein aglycone, and oleocanthal, among others [7,8]. Besides these prospective studies carried out with human populations, animal studies have also indicated that OO phenols have preventive actions on age-related cognitive and motor dysfunction. Furthermore, mechanistic *in vitro* studies also indicate that OO phenolic compounds may inhibit inflammatory pathways, induce signaling pathways related to cell protection and survival, and modulate pathways related to energy metabolism similar to anti-aging substances, which has been summarized in an excellent review [9]. The present review not only constitutes an update including the most recent advances on the research liking OO and the prevention of aging-related alterations, but also it is focused on the systematic evaluation of how OO and its minor constituents act on the recently recognized hallmarks of aging. Elucidating how OO targets these hallmarks can help us to better understand the molecular and cellular basis of its beneficial action on aging and aging-related diseases. Moreover, for our review we will consider not only OO (in its varieties) and the compounds contained in this edible oil at a pharmacologically relevant concentration, but also olive-related compounds that can be used as purified molecules or as extracts obtained from sources as olives and olive leafs, among others. These substances have been used for a significant part of the research aimed on giving a mechanistic explanation of how OO exerts its positive effects on cellular pathways that are altered with aging, and they can set up the basis for designing future olive-derived functional foods which positively impact human aging. 

## 2. Olive Oil and Genomic Instability

The genetic material tends to accumulate damage during aging because it is continuously challenged by exogenous and endogenous threats [1]. In an aging context, oxidative damage to mitochondrial DNA (mtDNA) is more important than the damage exerted to lipids and proteins due to the ability of mtDNA to be disseminated in the division of mitochondria and cells, which amplifies the physiological consequences of the damage [10]. Traditionally, mtDNA has been considered highly susceptible to oxidative attack because: (i) the mitochondrial respiratory chain is a source of a continuing flux of oxygen radicals; (ii) it is not protected by histones; and (iii) the mitochondria may be less efficient in repairing DNA damage and replication errors than the nucleus [11]. In addition, aging is associated with deletions of mtDNA in a tissue-dependent manner [12], affecting mainly postmitotic tissues like the brain, skeletal muscle and heart [13]. Some studies indicate that multiple mtDNA deletions may be promoted by double strand breaks [14].

Several studies have tried to test the effect of OO avoiding the age-associated damage in the DNA, both *in vivo* and *in vitro*. Quiles *et al.* have tested the effects of feeding male Wistar rats with diets containing different fat sources as VOO and sunflower oil (SO) [15]. Lower levels of DNA double-strand breaks in peripheral blood lymphocytes were found in young animals fed on VOO, which reached around one half of the damage that was found in the SO group. The same measurements were carried out in aged rats showing that, although the aging-related increase of DNA double-strand breaks levels that took place in both dietary groups, the damage was significantly lower in rats fed a diet containing VOO. However, the two groups did not differ in either mean or maximum life span.

Another study from the same research group was set to analyze the presence of a particular deletion in liver of rats fed diets containing VOO or SO [16]. Two regions of the mitochondrial genome were studied: ND1 and ND4 genes. The former is rarely affected by deletions in humans and rats whereas the latter is included in the so-called common deletion both in humans and rats. While an increase of more than 6-fold in the deletion was found in the case of old animals fed the VOO-containing diet, the increase was 60% higher (more than 10-fold) in old animals fed the diet containing SO. These observations support that dietary fat type can modulate the frequency of the studied deletion in rat liver and that the age-related increase in mtDNA deletions could be attenuated. The lower increase in mtDNA deletion frequency during aging was attributed to the lower amount of free radicals produced by VOO.

Fabiani *et al.* have measured the effect of OO phenolics on H_2_O_2_-induced DNA damage in human peripheral blood mononuclear cells (PBMC) and promyelocytic leukemia cells (HL-60) [17]. Their results showed that HT, a complex olive oil phenol extract (OOPE), and olive mill wastewater phenol extract (WW-PE) displayed a highly protective activity against DNA damage in both PBMC and HL-60 cells. Furthermore, other purified compounds like one isomer of oleuropein aglycon, oleuropein, tyrosol, caffeic acid and verbascoside also displayed a protective effect on cells although in a lower range. These results suggest that phenols used at low concentrations (1–10 mmol/L), as purified compounds or in a complex crude extract independently of the source (OO or WW-PE), may prevent DNA damage induced by H_2_O_2_. These concentrations could be easily reached in the tissues with an ordinary intake of 50 g/day of olive oil, because these phenolic compounds are effectively absorbed in humans [18].

Additional studies have demonstrated that pretreatment of Hela cells with OOPE also prevents nDNA damage induced by H_2_O_2_ [19]. This protection might be associated with OOPE’s free radical scavenging properties, metal ion chelating properties and/or the endogenous antioxidant defenses and DNA repair systems [17]. In addition, VOO phenolics could protect APEX1, a DNA repair gene, by inhibition of oxidative nDNA damage [19].

## 3. Olive Oil Effect on Telomere Attrition

Telomeres are nucleoprotein structures that protect the ends of eukaryote chromosomes [20]. Importantly, telomere shortening is also observed during normal aging both in human and in mice [21]. Telomere length is considered to be a biomarker of aging; shorter telomeres are associated with a decreased life expectancy and increased rates of developing age related chronic diseases [22,23,24]. However, telomere length varies considerably among individuals [25]. DNA in these structures is particularly susceptible to age-related deterioration [26]. One of the reasons for this is that replicative DNA polymerases lack the capacity to replicate completely the terminal ends of linear DNA molecules, a function that is proprietary of a specialized DNA polymerase known as telomerase. However, most mammalian somatic cells do not express telomerase, and this leads to the progressive and cumulative loss of telomere-protective sequences from chromosome ends [1]. Moreover, telomeres are bound by a characteristic multiprotein complex known as shelterin [27] that prevent the access of DNA repair proteins to the telomeres. Although telomere length is strongly influenced by genetic factors, telomere attrition might be modulated by environmental and lifestyle factors, including older age, cigarette smoking, gender, ethnicity [28], education [29] and other indicators of socioeconomic status [30] and diet [31]. Studies have suggested that telomere attrition is modifiable, as substantial variability exists in the rate of telomere shortening that is independent of chronological age [32,33,34]. Therefore, variability of telomere length may be partially explained by lifestyle practices, including dietary patterns.

Some reports have indicated a positive relationship of leukocyte telomere length and Mediterrranean diet adherence in two different human population subsets [35,36]. These included a cohort of elderly subjects from the South Italy [35] and a group of nurses aged 30–55 years [35]. Similarly, a study comparing between Greek and Dutch elderly men has reported that the latter presented lower leukocyte telomere length [37]. When measured, telomerase activity has also shown a positive association that correlates with leukocyte telomere length [35]. Oxidative stress may influence the rate of telomeres shortening [37]. In that sense, it has been reported that telomerase activity was negatively modulated by inflammation and oxidative stress [35]. In contrast, no relationship between leukocyte telomere length and indicators of oxidative stress and plasma antioxidants was found in the study comparing Dutch and Greek elderly men, although the endogenous serum antioxidants albumin and uric acid were positively associated with telomere length [37].

Studies directly evaluating the effects of OO intake on leukocyte telomere length are absent. Of note, regression analysis showed that the Mediterranean diet adherence was not associated with leukocyte telomere length in the overall study population after adjusting for age, sex, education, ethnicity, caloric intake, smoking, and physical and leisure activities. However, a significant association with leukocyte telomere length was recognized among non-Hispanic whites. Furthermore, it was found that higher adherence to a Mediterranean diet was associated with longer leukocyte telomere length among whites, but not among African Americans and Hispanics [38]. Thus, results may not be generalized to other populations.

Another intervention has been performed on elderly subjects that consumed three diets, each for four weeks: a saturated fatty acid (SFA) diet, a low-fat and high-carbohydrate diet, and a Mediterrranean diet enriched in MUFA following a randomized crossover design. Human umbilical endothelial cells were incubated with serum from each patient. Among these, the MUFA-rich diet induced lower percentage of cells with telomere shortening, compared with the baseline and the other two diets. Moreover, this effect correlated with lower intracellular production of reactive oxygen species (ROS) and diminished cellular apoptosis [39].

The *Prevención con Dieta Mediterránea* (PREDIMED) trial consists in a 5-year nutritional intervention with three groups: Mediterranean diet specifically supplemented with EVOO, Mediterranean diet supplemented with mixed nuts, or a low-fat diet as control group. In a subset from this study, higher baseline telomere length significantly predicted a greater decrease in body weight body mass index (BMI), waist circumference and waist to height ratio. In addition, changes in telomere length during the 5-year intervention were inversely associated with changes in the four anthropometric variables. The reduction in adiposity indices during the intervention that was associated with increased telomere length was even higher among subjects with the longest telomeres at baseline. Logistic regression analysis showed that the risk of remaining obese after 5 years was lower in those participants who initially had the longest telomeres and increased their telomere length after intervention [40].

Telomerase itself could be a potential target to prevent telomere length attrition. Actually, telomerase deficiency in humans has associated with premature development of diseases, such as pulmonary fibrosis, dyskeratosis congenita, and aplastic anemia, which involve the loss of the regenerative capacity of different tissues [41]. Two phenolic compounds, oleacein and oleuropein, mainly present in olive leaves, seem interesting at this respect according to the results obtained in an *in vitro* assay. Namely, exposition of endothelial progenitor cells to any of these compounds prior to angiotensin II treatment increased telomerase activity. This effect was also associated with an increase of proliferation and a decrease in the percentage of senescent cells and intracellular ROS formation [42], as described in the “Stem Cell Exhaustion” section. However, it has to be taken into account that the amount of oleuropein in EVOO is very low. Furthermore, whether OO intake may affect telomerase activity is still poorly investigated and no *in vivo* effects have been reported, which warrants future research.

## 4. Epigenetic Changes Induced by Olive Oil

Epigenetic modifications are heritable changes in gene function that are not caused by variation in DNA sequence [43]. These changes mainly involve alterations in DNA methylation patterns, posttranslational modification of histones, and chromatin remodeling. There are multiple enzymatic systems assuring the generation and maintenance of epigenetic patterns, including DNA methyltransferases, histone acetylases, deacetylases, methylases, and demethylases, as well as protein complexes implicated in chromatin remodeling. Aging-associated transcriptional signatures also affect noncoding RNAs, including microRNAs (miRNAs) [1]. Epigenetic processes are dynamic and may be affected by environmental factors such as exercise and diet [44,45]. Actually, diet is the more studied environmental factor in epigenetics [46].

One of the most common epigenetic marks is DNA methylation. DNA methylation may play an important role in causing disease by silencing genes through hypermethylation or activating genes through hypomethylation. Cytosine DNA methylation, the most studied epigenetic mechanism, is usually considered as a flexible method for repressing gene expression. Different dietary and lifestyle factors are able to modulate the methylation of specific CpG sites in gene promoters even in the adult age [45]. Changes have been observed in DNA methylation patterns during aging [47,48], although hypermethylation has been noted in some loci while hypomethylation has been also noted in others [49].

In relation to OO, an observational study performed on Greek adolescents has been recently published with very interesting results. A genome-wide methylation profile in blood revealed that the MUFA to SFA ratio in the diet was associated with 26 island shores and 158 site [50]. This dietary feature is typical of Mediterranean diets, likely due to the high intake of OO [51]. In that sense, dietary interventions with Mediterranean diets have also shown that DNA methylation levels were associated with adherence to the diet [52]. According to previous findings, it is possible that OO was able to induce epigenetic changes by means of its MUFA content. However, an *in vitro* study carried out with human colon cancer cells (Caco-2) has reported very similar effects for VOO and its phenolic compounds [46].

Along with DNA methylations, EVOO could also induce epigenetic changes by histone acetylation processes. In support of this, an *in vitro* assay has shown that some phenolic compounds contained in EVOO, namely secoiridoids (*i.e.*, oleuropein and its conjugated forms), are able to induce hyperacetylation of histone H3 in cell cultures, among many other effects [53].

Another point of interest is the possible genes affected by methylation and their implications for aging process. All the epigenetic changes derived from OO or its minor compounds have been usually studied only in specific genes, and several of these genes have shown relationship with the aging process. In particular, many of them have been related with susceptibility to aging-related diseases such as metabolic syndrome. In relation to this, some intervention studies have indicated that OO or Mediterranean diets influence DNA methylation in genes encoding enzymes involved in fatty acid metabolism. In renal patients, OO supplements altered methylation of fatty acid desaturase (FADS)-2 CpGs and elongase (ELOVL)-5 CpGs. The methylation status of the altered CpGs in FADS2 and ELOVL5 was associated negatively with the level of their transcripts [54]. Similarly, in a dietary intervention study with Mediterranean diet to decrease weight of the subjects, those who lost weight showed higher levels of stearoyl CoA desaturase (SCD)-1 gene promoter methylation after one year. Furthermore, these subjects presented higher adherence to a Mediterranean diet [52]. Previously, it was reported that variations in SCD activity were associated with disorders such as obesity, insulin resistance, and diabetes [55,56], which suggests that gene expression modifications can affect metabolic syndrome onset and progression. In addition, it has been reported that SCD1 knockdown increased the amount of SFA and decreased that of MUFA in HeLa cells membrane phospholipids without affecting the amount or the composition of free fatty acids [57]. The degree of fatty acid unsaturation in membrane phospholipids affects many membrane-associated functions which highlights the importance of SCD1. In particular, OO intake increases membrane MUFA content [58], which could be accounted not only by its high-MUFA content but also by its effect on SCD1 gene, reinforcing the notion that Mediterranean diet effect on SCD1 may be a consequence of OO. 

Other genes interesting in relation to metabolic syndrome are those involved in circadian rhythms. In women, it has been reported that the percentage of methylation of the clock circadian regulator gene (CLOCK) CLOCK CpGs 1 and 8 showed associations with the intake of MUFA and polyunsaturated fatty acids (PUFA) [59]. The circadian clock system instructs 24 h rhythmicity on gene expression in essentially all cells [59] and it has been related to obesity and metabolic syndrome features. It has been reported that patients with visceral obesity and metabolic syndrome exhibit disturbances in the circadian rhythm (chronodisruption) that may be associated with higher weight increase and development of diabetes and atherosclerotic disease [60,61]. This is in accordance with findings from the same study since a different pattern of DNA methylation of CLOCK and BMAL1 gene between normal-weight and overweight or obese subjects was noted. Moreover, the methylation pattern of different CpG sites of the three genes showed significant associations with anthropometric parameters such as body mass index and adiposity, and with a metabolic syndrome score [59].

On the other hand, an *in vitro* study with Caco-2 cells has also shown possible anticancer effects for EVOO by means of this mechanism, since it influenced DNA methylation state at CNR1 promoter which was inversely correlated to selective and transient up-regulation of CNR1 gene, a tumor suppressor that encodes for type 1 cannabinoid receptor (CB1). EVOO, a phenolic EVOO extract or HT stimulated CB1 expression which was associated with reduced proliferation of these cancer cells [46]. The same authors confirmed this anticancer effect in rats where dietary EVOO supplementation increased CB1 mRNA. Consistently, CpG methylation of rat Cnr1 promoter, miR23a and miR-301a, previously shown to be involved in the pathogenesis of colorectal cancer [62] and predicted to target CB1 mRNA, were reduced after administration of EVOO down to ~50% of controls [46]. In another *in vitro* study, EVOO phenolic extracts rich in secoiridoids permitted Histone H3 remain in hyperacetylated state at Hysine 18 in a breast cancer model (the HER2-gene amplified JIMT-1 cell line, a unique breast cancer model). The resulting alterations in gene regulation could reduce mitotic viability and metabolic competence of breast cancer cells, inherently refractory to HER-targeting therapies *ab initio* [53].

miRNAs are small endogenous non-coding RNAs that regulate several cellular and biologic processes by regulating gene expression [63] that may epigenetically explain the long-term phenotypic changes of the offspring. Some researchers have also studied this mechanism deepened in. In rats, it has been reported that maternal consumption of different types of fat sources, including OO, during early pregnancy influenced miRNAs expression in both maternal and offspring tissues [64]. The influence of dietary fat on miRNAs has also been reported in humans. In the PREDIMED randomized trial, a novel association between a microRNA target site variant and stroke incidence was reported which is modulated by diet in terms of decreasing triglycerides and possibly stroke risk in rs13702C allele carriers after a highly-unsaturated fat Mediterranean diet intervention [65].

## 5. Olive Oil Effects on Proteostasis

A multitude of quality control mechanisms have evolved to preserve the stability and functionality of proteomes in cells. These mechanisms function in a coordinated fashion to restore the structure of misfolded polypeptides or to remove and degrade them completely, thus preventing the accumulation of damaged components and assuring the continuous renewal of intracellular proteins [1]. Protein homeostasis or proteostasis involves both mechanisms for the stabilization of correctly folded proteins (*i.e.*, the heat shock family of proteins) and mechanisms for the degradation of proteins (*i.e.*, the ubiquitin-proteasome system and those including lysosomes) [66,67,68]. The heat shock family of proteins includes several isoforms of 70-kDa heat shock proteins (Hsp70) that can be constitutively expressed and stress-induced as well. They are chaperones involved in crucial cellular functions [69,70,71,72]. While constitutively expressed Hsp70 chaperones have housekeeping functions (e.g., folding of nascent polypeptides, protein translocation between cellular compartments and degradation of unstable and misfolded proteins), stress-induced Hsp70s prevent the accumulation of proteins that have become denatured in response to various cellular stresses (e.g., heat stress, radiation, ischemia, heavy metals or other stimuli that activate stress transcription factors). Stress-induced synthesis of cytosolic and organelle-specific chaperones is significantly impaired in aging [73] and research with animal models supports the positive impact of chaperones on longevity [74,75,76,77].

Aging and some aging-related diseases are associated with perturbed proteostasis, and its experimental perturbation can precipitate age-associated pathologies [1,67,78]. This is particularly notably in some age-related pathologies, such as Alzheimer’s disease, Parkinson’s disease, and cataracts where chronic expression of unfolded, misfolded, or aggregated proteins contributes to their development [78]. Different *in vitro* studies have shown interesting activities of olive phenolic compounds on protein aggregates of this type. HT, oleuropein, and oleuropein aglycone prevented Tau fibrillization, a major feature in Alzheimer’s disease (AD) pathogenesis, in an *in vitro* assay [79]. In Type II diabetes, pancreatic amyloid deposits of amylin are apparently cytotoxic to β-cells. The main phenolic component of EVOO, oleuropein aglycone, when present during the aggregation of amylin, consistently prevented its cytotoxicity to RIN-5F pancreatic β-cells. Moreover, a lack of interaction with the cell membrane of amylin aggregates was observed when cells were grown in the presence of oleuropein. These observations suggest that oleuropein aglycone interferes with amylin aggregation, resulting in a different path skipping the formation of toxic prefibrillar aggregates [80]. Therefore, it is possible that EVOO could be used to prevent or treat these aging-related diseases or aging changes possibly due to some compound present in this food having an effect on a mechanism regulating protein homeostasis. The relationship between EVOO and AD will be considered specifically in a later section (see Section 8: Olive oil and Cellular Senescence).

Different compounds or extracts from EVOO have been related with an increase of some components of the chaperones family or up-stream factors regulating them. Treatment with an EVOO secoiridoid-rich phenolic extract markedly up-regulated the expression of the HSPA6 (Hsp70B’) gene, which is strictly inducible with no detectable basal expression [81,82]. Indeed, HSPA6 protein induction is a sensitive biomarker of cellular stress that appears transiently in response to heat stress, whereas levels of HSPA1A (Hsp72), which was also induced by an EVOO secoiridoid-rich phenolic extract, persist for days [83]. The secoiridoids also enhanced the expression of the HSPA1L gene (Hsp70-hom or Hsp70t), which encodes a constitutively expressed, non-inducible cytosolic protein that is highly abundant in testis [84,85], and of HSPA2 (Hsp70-2), a constitutively expressed gene that is also expressed at high levels in testis [86]. Treatment with tyrosol expanded longevity of several mutant strains of *C. elegans* and such effect has been related with the master regulator of the heat-shock response, the transcription factor HSF-1 [87]. The importance of the activation degree of this transcription factor for longevity and thermotolerance increase in nematodes has been previously reported [88,89]. In mammalian cells, deacetylation of HSF-1 by SIRT1 potentiates the transactivation of heat-shock genes such as Hsp70, whereas down-regulation of SIRT1 attenuates the heat-shock response [90]. SIRT1 interacts with FOXO1, an anti-aging transcription factor which is negatively regulated by Akt [91,92]. OO could have effect on proteostasis by means of SIRT1 activation. In skeletal muscle cells, treatment with oleic acid (OA) increased intracellular levels of cyclic adenosine monophosphate (cAMP) that turned on protein kinase A activity. This resulted in SIRT1 phosphorylation at Ser-434 and elevation of its catalytic deacetylase activity [93].

In relation to proteasome, continuous treatment with oleuropein has shown to increase proteasome-mediated degradation rates in early passage human embryonic fibroblasts cultures, possibly through conformational changes of the proteasome. As consequence, a decrease in the amount of oxidized proteins was also observed. In addition, oleuropein-treated cells retained proteasome function during replicative senescence and cultures exhibited a delay in the appearance of senescence morphology and their life span was extended by approximately 15% [94] which confirmed the importance of proteasome function in aging processes. Furthermore, this indicates that certain extracts from olive leaf may be beneficial with respect to proteasome activity. The possibility exists that oleuropein derivatives found in greater concentration in EVOO might exert similar effects, which remains for further investigation. Indeed, there are more compounds shown to influence expression of factors implicated in cell signaling pathways that contain components of the proteasome-ubiquitin system. Regarding autophagy, it has been reported that EVOO feeding increased mRNA levels of the authophagy marker LC3 with respect to other dietary fat sources (SO and fish oil) in 24 months old rat gums. In the same study, levels of mRNA were also measured in rats aged 6 months, but LC3 change was only clearly noted in old animals [95]. In turn, dietary supplementation with EVOO decreased mRNAs levels of the autophagy markers LC3 and Beclin1 in muscle of a transgenic mouse model of amyotrophic lateral sclerosis, among other effects [96]. These results are consistent with the idea that VOO targets other processes that prevent protein damage in this tissue and, consequently, less protein removal is needed.

Research focused on the effects of different compounds on cell signaling cascades has also supplied interesting results concerning autophagy control. In particular, the mammalian target of rapamycin (mTOR) have spurred extraordinary interest after the demonstration that constant or intermittent administration of its inhibitor, rapamycin, can increase the lifespan of middle-aged mice [97,98] and delays multiple aspects of aging in mice [99]. In most of cases, the actions of OO compounds on mTOR-dependent signaling were accompanied by higher surveillance and stress resistance. Part of these effects also could be caused by effects on other processes regardless of autophagy, as many antioxidant defense systems share part of these signaling cascades [100]. Since mTOR route is specifically involved in nutrient sensing, another well-established hallmark of aging, the effect of OO components on the mTOR kinase will be described in the next section (see Section 6: Olive oil and Nutrient Sensing Pathways).

The effects of fatty acids and other minor compounds from EVOO on the unfolded protein response (UPR), a specific pathway aimed to restore proteostais in endoplasmic reticulum (ER), have been also extensively studied. This pathway mainly operates by attenuating protein synthesis and import to ER, and by activating a cascade of transcription factors that regulate genes encoding for ER chaperones, components of the ER-associated protein degradation system (*i.e.*, ubiquitin-proteasome components), and components of the autophagy machinery [101,102]. As in other cell compartments, unfolded or misfolded proteins can accumulate in the ER lumen, aggregate and hence become toxic and detrimental to cell survival [101]. Facing these situations collectively referred as ER stress, cells have evolved systems to detect, eliminate and avoid further accumulation of unfolded or misfolded proteins. ER stress seems to occur in correlation with senescence, leading to activation of UPR [101]. Actually, some chronic diseases have been associated with apoptosis or necrosis derived from ER stress including diabetes mellitus [103] or diabetic nephropathy [104].

Two *in vitro* studies have shown that, in comparison to SFA, MUFA as present in OO could prevent or decrease apoptosis and/or necrosis derived from ER stress [103,104]. In INS-1E β-cells, combination of oleate with palmitate prevents apoptosis and viability decrease that is otherwise noted when cells are treated with palmitate alone. In addition, oleate also prevents the increase of UPR marker associated to palmitate, which suggested that oleate prevent ER-stress and thus, UPR is not needed. On the other hand, mRNA expression of the ER chaperones (Bip, Pdi, Calnexin and Grp94) was not altered by fatty acids in this model [103]. This suggests that oleate protects INS-1E β-cells from palmitate-induced apoptosis by the suppression of ER stress which was independent on chaperone activation. The analysis of the data from different studies does not highlight any differences regarding the cell type [101]. The activation of the UPR seems to occur in all types of senescence, whatever the inducer is successive replications [105,106], oncogene activation [107], DNA-damaging agents [107,108], or oxidative stress [105]. However, the precise signature of ER stress varies according to the context [101]. In the same sense, analysis of SCD1 knockdown models that lead to an increase of SFA and a decrease of MUFA have been associated to several markers of UPR activation in different cell cultures [57]. In other study carried out at a cellular level, such intervention has shown to increase palmitate-induced ER stress and apoptosis, whereas overexpression of SCD2 that increased desaturation of palmitate to MUFA, attenuated palmitate-induced ER stress and apoptosis [109].

According to these findings, a decrease in membrane MUFA would induce ER stress and, as a consequence, UPR would be activated to restore homeostasis. On the contrary, an increase of MUFA makes cells less susceptible to SFA-induced ER stress and apoptosis. OO has been related to an increase of MUFA membrane content [58]. Thus, based on the fatty acids profile in OO, this food should decrease accumulation of misfolded and unfolded proteins in ER and possibly in other organelles, although the mechanisms under this effect need to be explained. Similarly, there are also *in vitro* evidences for effects of other minor compounds on this sense. An EVOO secoiridoid-rich extract upregulated the transcriptional expression of the DNAJA4 and DNAJC3 genes, which encodes the ER-localized DNAJ family of proteins [110]. The induced transcription of DNAJ genes is part of the UPR [111,112,113,114]. The ability of secoiridoids from EVOO to upregulate a set of genes involved in the ER stress response to accumulation of unfolded proteins may appear to conflict with the demonstrated ability of these compounds to strongly inhibit the growth of highly aggressive breast cancer cells [53,115]. However, the UPR is the major protective and compensatory mechanism that enables cells to survive during ER stress. Secoiridoids promote cell death-UPR branch signaling by impeding the alleviation of ER stress [116].

## 6. Olive Oil and Nutrient Sensing Pathways

Aging is characterized by a deterioration of homeostatic maintenance over time, leading to a functional decline with increased risk for disease and death. This process is metabolically characterized by insulin resistance, changes in body composition, and physiological declines in sex steroids, growth hormone (GH) and insulin-like growth factor-1 (IGF-1) [117]. This latter alteration is indicative of a deregulation in a nutrient sensing route during aging, because the intracellular signaling pathway of IGF-1 is shared with that of insulin, which informs cells of the presence of the glucose, being then regarded as insulin/IGF-1 signaling (IIS) [1]. Genetic manipulations carried out in different animal models as worms, flies and mice in order to down-regulate this route have resulted in extended lifespan [118] but, paradoxically, levels of GH and IGF-1 show a decline during both normal and premature aging [119]. This apparent paradox can be explained on the basis of a defense response elicited by the organism in order to minimize cell growth and metabolism in a systemic damage scene [120].

Since high levels of IGF-1 are often associated with different kinds of cancer, a defensive response against damage may have the risk of becoming deleterious and aggravating aging. In accordance, low peripheral IGF-1 levels are associated with increased risk of numerous pathologic conditions in aging humans, including type 2 diabetes mellitus, cardiovascular diseases, sarcopenia, osteoporosis, and frailty [117,121]. Many research groups have thus tried to palliate these deleterious alterations using natural substances such as OO and its derivatives. In this way, González and colleagues [122] first postulated the antidiabetic effect of oleuropein and HT in an animal model of alloxan-induced diabetes mellitus. Similar studies have been performed by other groups that strongly support this protective effect. Al-Azzawie and Alhamdani [123] used 3 months-old New Zealand male rabbits rendered diabetic by alloxan treatment and a group of these animals were given a daily oral dose of 20 mg/kg of oleuropein for 16 weeks. Blood glucose levels were decreased in oleuropein-treated group in comparison with the diabetic controls. An increase in oxidative stress was shown in all diabetic rabbits but was decreased significantly by oleuropein after 16 weeks of treatment. In accordance, enzymatic and non-enzymatic antioxidants were diminished after diabetes induction, but oleuropein treatment reverted this effect, supporting that oleuropein attenuated oxidative stress and enhanced body´s own antioxidant defenses.

Hamden and partners [124] tested the hypoglucemic and antioxidant effects of purified HT and WW-PE, both in alloxan-induced diabetic rats treated for two months and through *in vitro* models. Phenolics were extracted from olive mill waste to separate two fractions: one of them enriched in monomeric phenolics (F1) and the other one enriched in oligomeric and polymeric phenolics (F2). Glucose levels were diminished with all the treatments whereas hepatic glycogen levels were increased with respect the diabetic group. An improvement of antioxidant defense and a decrease in hepatic and renal index of toxicity were observed in the presence of the OO phenolics in comparison with the diabetic non supplemented group. The histological analyses revealed the reduction of fatty cysts in liver, the decrease of fatty infiltration in kidney, and the improvement of vascular degenerative damage and number of β-cells in pancreas, being these observations indicative of tissue protection against deleterious diabetic alterations. *In vitro* assays were carried out in parallel in perfused pancreas of non-diabetic rats with different concentrations of glucose plus F1, F2 or HT treatment. Of note, when pancreatic tissue was incubated with high levels of glucose combined with monomeric phenols or HT, the organ increased insulin secretion, which was otherwise repressed when maintained with high-glucose without additional treatment. Following a similar approach, another research group tested the antidiabetic and antioxidant activity of oleuropein and HT at 8 and 16 mg/kg of body weight in alloxan-induced diabetic Wistar rats [125]. Results indicated that polyphenols administration significantly decreased blood glucose and increased hepatic glycogen levels. As reported previously, polyphenols attenuated oxidative stress and restored antioxidant defense systems that had been down-regulated in diabetic rats. In addition, these beneficial effects followed a dose dependent response, with the highest concentrations of polyphenols showing the most prominent effects.

More recently, de Bock and collaborators carried out a 30-week randomized, double-blinded, placebo controlled, crossover trial [126] in order to test the effects of supplementation with an olive leaf extract (OLE) in middle-aged overweight men, who are more prone to be insulin resistant. Participants received capsules with OLE or placebo for 12 weeks, and they changed the treatment after 6 weeks of washout period. Interestingly, OLE supplementation improved insulin sensitivity by 15% and the pancreatic β-cells function by 28%. This is consistent with the decreased levels of glucose and insulin reached in blood. Higher levels of interleukin-6 and IGFBP-1 and -2 were observed, while liver function, lipid profile and carotid intima media thickness did not show any changes among OLE *vs.* placebo participants. Of note, this study demonstrated that two aspects of glucose regulation, pancreatic β-cell function and insulin sensitivity, were improved in just 12 weeks of treatment.

Besides the IIS, changes in other nutrient-sensing systems are also associated with aging process. One of best characterized systems is the mTOR kinase pathway, which plays a role in sensing high amino acid concentrations in the organism [1]. Genetic down-regulation of one of the complexes which constitute the mTOR kinase, mTORC1, extends longevity in yeast, worms and flies [127]. Studies carried out by Harrison *et al*. [128] in mice using rapamycin, a well-known inhibitor of mTOR, have shown extended longevity and are considered as the most robust chemical intervention to increase lifespan in mammals [98]. Conversely, the abnormal activation of this serine/threonine kinase is associated with a decrease in lifespan and, furthermore, is frequently observed in diseases associated with aging, such as cancer [128,129], Alzheimer´s disease [130,131] and diabetes [132].

Several research groups have tested different kind of substances able to inhibit mTOR kinase, considered as a good target for the treatment of cancer [133]. Evidences from independent studies carried out with cell cultures have indicated that compounds contained in EVOO could target differentially mTOR signaling cascades. On the one hand, HT was shown to activate the mTOR/p70S6-kinase pathways in cultured human retinal pigment epithelial cells from the ARPE-19 cell line [100], and a gene expression study reported that OA increased mTOR pathway genes expression in preadipocytes cultures [134]. On the other hand, oleocanthal, which exhibits potent anticancer and neuroprotective activities, inhibited the enzymatic activity of mTOR in other *in vitro* assay, and the proposal was made that beneficial activity of oleocanthal might be due, at least in part, to mTOR inhibition [135]. Inhibitory effects on mTOR activity by different concentrations of oleocanthal were clearly observed, which led to antiproliferative effects in MCF-7, T47D and MDA-MB-231 breast cancer cell lines, with the latter cells being the most affected. However poor antiproliferative effects were shown in colorectal (Caco-2) and cervical (HeLa) cancer cell lines. This could be due to the fact that mTOR is highly expressed in breast cancer cell lines. In addition, analyses of HGF-induced mTOR phosphorylation were carried out in MDA-MB-231 cells, either in the absence or in the presence of oleocanthal, and the results indicated that this secoiridoid was able to suppress mTOR phosphorylation by more than 60% without altering its total levels. In sum, these evidences strongly support the idea that OO, and particularly oleocanthal, can regulate the altered levels of mTOR activity in order to protect the organism against diseases as cancer.

## 7. Mitochondrial Dysfunction

The free radical theory of aging proposed by Harman [136] is based on the accumulation of oxidative damage in cells and tissues over time and its contribution to the decline of physiologic function with age. Although multiple data support an important role for ROS in aging, developments of the last years have led to a re-evaluation of this theory [1,137]. Leaving aside their deleterious role in aging, it is clear that ROS contribute in triggering proliferative and survival pathways in response to physiological signals and stress conditions [138]. Both evidences could be harmonized if we take into account a dual role for ROS: first, as activators of compensatory homeostatic responses which increase during aging in order to maintain survival and second, as factors that aggravate aging-associated damage when their levels reach a certain threshold [137]. Related with this second role, mitochondria have been proposed as an important link between oxidative damage caused by ROS and lack of normal function. The electron transport chain located in its inner membrane (METC) metabolizes the majority of the total oxygen taken by cells, and this organelle is thus exposed to by-products as superoxide anion radicals or hydrogen peroxide which are potential sources of intracellular damage. This fact makes the mitochondria a major target of oxidative damage which can lead to reduced energy production and a compromised cell function [10].

Double bonds of membrane fatty acids are particularly sensitive to oxidative attack, which restricts the normal ability of molecules to diffuse thus modifying membrane fluidity and finally affecting the their normal function [10]. It is well known that the predominant fat source of the diet influences biochemical parameters of mitochondrial membranes [139] because these organelles adapt fatty-acid composition profile and electron-transport systems in relation to dietary fat. In that sense, compared with a saturated or a monounsaturated dietary fat source, a polyunsaturated fat source leads to membranes that are more prone to oxidation [140]. For this reason, several groups have focused their research on the potential protective role of OO during aging. 

Quiles and colleagues carried out a study in order to assess how the electron-transport components are affected in several tissues by feeding Wistar rats throughout their lifespan with two different dietary fat sources: SO (enriched in n-6 PUFA) and OO (enriched in n-9 MUFA) [141]. Mitochondrial-lipid profile, concentration of coenzyme Q (CoQ), cytochrome *b* and complex IV (cytochrome *c*-oxidase) activity and turnover were analyzed in liver (mitotic tissue), heart and skeletal muscle (post-mitotic tissues) from rats fed experimental diets for 6 or 24 months. The most profound effects of age were found in postmitotic tissues, which exhibited an overall increase in CoQ and cytochrome *b* together with a decrease in the activity and turnover of complex IV. In another complementary study [142], the mitochondrial fatty-acid profile as well as the levels of mitochondrial hydroperoxides, CoQ, α-tocopherol, and catalase activity were measured in liver, heart and skeletal muscle from Wistar rats fed diets containing the same fat sources (*i.e.*, SO *vs.* OO) for 6, 12, 18 or 24 months and a lipid source-dependent adaptation of mitochondrial fatty-acid profile was clearly observed. Animals fed on a diet containing OO showed the highest amount of MUFA in their mitochondrial membranes, while animals fed on a diet containing SO displayed the highest amount of n-6 PUFA and these changes were in accordance with levels of oxidative damage. Among the three tissues studied, liver showed the lowest amount of hydroperoxides. Heart and skeletal muscle hydroperoxides increased with aging in both dietary groups, although animals fed with OO showed lower hydroperoxides than those fed with SO at all ages. These observations indicate that an OO-containing diet leads to less polyunsaturated membranes, resulting in the attenuation of aging-related increase of lipid peroxidation in post-mitotic tissues, the most relevant tissues in aging [143].

Interestingly, levels of several antioxidant molecules changed in a differential way depending on the tissue regenerative capacity. In this way, concentration of α-tocopherol decreased in aging liver in both dietary groups, but increased in heart and skeletal muscle. CoQ levels followed a similar pattern, being higher in post mitotic tissues. This could be interpreted on the basis of a greater necessity to accumulate these antioxidants in tissues that could elicit a more intensive response against oxidative damage [144]. An increase in catalase activity is associated with greater resistance against oxidative damage, and this seems to be crucial in overall antioxidant enzymatic defense with respect to the lifespan [145,146]. Thus, in liver, this activity was increased with aging in rats fed the OO-containing diet, but continued unchanged in animals fed the SO-containing diet. In heart, although both dietary groups showed an increment in catalase activity with aging, the increase was higher in the OO group. However, in skeletal muscle catalase activity decreased with aging in both dietary groups.

Taken together, these previous studies suggest that regenerative capacity is a plausible factor to explain how dietary lipids can modulate aging in different tissues. In accordance with this interpretation, the mitochondria suffer oxidative alterations and damage with aging [143,145] and these alterations can deteriorate mitochondrial structure and function depending on the tissue capacity to repair or to remove cell damage. Regenerative tissues, as it is the case of liver, would be able to buffer this damage, as suggested by the lack of cytochrome oxidase activity loss in liver. However, postmitotic tissues, as heart and skeletal muscle, are not capable to remove damaged cells leading to decreased cytochrome oxidase activity which could produce an uncoupling of the METC and, subsequently, an increase in free radical production [142]. Interestingly, another study carried out with skeletal muscle of old rats fed an OO-containing diet for 6 weeks demonstrated that the aging-related loss of METC enzyme activities, particularly complex I (NADH-CoQ oxidoreductase) and complex IV (cytochrome *c*-oxidase), could be restored by this edible oil to reach the activity levels of young animals [147].

Overall, these results highlight the important role played by dietary lipid sources in the modification of mitochondrial membrane profile in order to generate a more “safe environment” against the oxidative damage and lack of function associated to aging, with the MUFA-rich OO being an optimal lipid source to perform this beneficial effect.

## 8. Olive Oil and Cellular Senescence

In 2012 a meta-analysis of more than 300 genome wide association studies (GWAS) identified the INK4a/ARF locus as the genomic locus that is genetically linked to the highest number of age-associated pathologies, including several types of cardiovascular diseases, diabetes, glaucoma, and AD [1,148]. This last disease is a neurodegenerative pathology that affects about 30 million people worldwide [149]. Several epidemiological studies indicate that the prevalence of AD and cognitive decline is low among the Mediterranean area populations compared with other geographical regions of the world [150,151,152]. Since phenolic constituents of EVOO as oleocanthal, oleuropein or HT have shown antioxidant and anti-inflammatory effects [153,154,155], many research groups are focusing their studies in elucidating the molecular mechanisms underlying the prevention or amelioration of neurodegenerative diseases by this edible oil [155,156].

Although a complex disease, two well-defined phenomena are recognized in AD: the amyloid-related neuropathological alterations in the brain, due to accumulation and deposition of amyloid-β (Aβ) peptide [157] derived from proteolysis of Aβ precursor protein (APP) [158], and the tau-related neuropathological alterations which results in intracellular insoluble neurofibrillary tangles [159]. Two principal processing APP pathways have been identified: the amyloidogenic route which leads to Aβ generation and whose molecular marker is sAPPβ, and the non-amyloidogenic route which prevents the Aβ generation whose indicator is sAPPα [160,161]. On the other hand, tau is a protein involved in the stabilization of the microtubules by direct interaction with a microtubule-binding domain (MBD), working as a modifier of the cytoskeleton plasticity [162,163]. In brain from AD patients this protein is hyper-phosphorylated and then, its binding to microtubules is inhibited [159,164]. Under these conditions, tau dissociates from axonal microtubules and aggregates itself in an abnormal way to create insoluble paired helical filaments implicated in neurodegeneration [165].

Oleocanthal has been suggested to reduce the polymerization of tau protein through a covalent mechanism [166,167]. Monti and collaborators [168] studied the recognition process and the reaction profile between this phenolic compound and the wild-type tau protein, tau-441. Their results showed that the interaction between them was covalent and nonspecific. This interaction induced a conformational rearrangement of the protein due to a fast transition of the tau-441 secondary structure to random coil to α-helix. This reorganization could explain the antifibrillogenic effect of oleocanthal and at least the down-regulation of the pathological phosphorylation of tau. In 2013, Abuznait and colleagues [169] reported one study focused on the role of oleocanthal in both *in vitro* and *in vivo* models of neurodegeneration. BEnd3 cells, a mouse brain endothelial line, were treated with different concentrations of oleocanthal ranging from 0.5 to 50 µM. On the other hand, the *in vivo* model consisted in C57BL/6 wild-type mice treated with oleocanthal (10 mg/kg) during 2 weeks. The accumulation of Aβ and its clearance from the brain facilitated by two major transport proteins: P-glycoprotein (P-gp) and low density lipoprotein receptor-related protein-1 (LRP1) at the blood-brain barrier were analyzed. The accumulation of Aβ in these cells were studied by uptake assays using a radio-labeled peptide ^125^I-Aβ40, which has been implicated in the pathogenesis of AD [161]. P-gp limits the entry of ^125^I-Aβ40 due to its efflux function whereas LRP1 enhances the cellular uptake of ^125^I-Aβ40. In addition, the potential modifications in Aβ degradation were also studied by analyzing the levels of two enzymes implicated in the process: insulin degrading enzyme (IDE) and neprilysin (NEP). *In vitro* results showed a significant increase of both transport proteins, P-gp and LRP1, in the presence of oleocanthal when compared to control untreated cells. However, specific roles for P-gp and LRP1 and their up-regulation in the presence of oleocanthal were recognized. Function of P-gp was enhanced in the presence of oleocanthal because cellular uptake of ^125^I-Aβ40 was significantly increased in the presence of the P-gp inhibitor verapamil (100 µM). In contrast, treatment of cells with the LRP1 inhibitor RAP (1 µM) in the presence of oleocanthal slightly decreased the uptake, although to a lower extent than in control cells, suggesting that oleocanthal might induce an unknown uptake mechanism that is independent of LRP1 and not inhibited by RAP.

The Aβ clearance from the brain can proceed through both saturable and nonsaturable routes. The saturable pathway involves degradation (via IDE and NEP) and efflux (via P-gp and LRP1). The brain efflux index (BEI%) method was used to determine ^125^I-Aβ40 clearance through the saturable pathway. In the presence of oleocanthal, the removal process was stimulated. This improvement was specifically enhanced by an increment in P-gp and LRP1, because in the presence of inhibitors, valspodar (24 ng/0.5 µL injection) and RAP (19.5 ng/0.5 µL injection) respectively, a reduction in BEI% was observed. The percent of degraded Aβ peptides was higher in the treated group, as well as the levels of IDE and NEP. Is sum, this study confirms that oleocanthal is able to up-regulate the expression of proteins which lead a better clearance and degradation of pathogenic molecules involved in AD.

Very recently, the same research group has carried out another study set to investigate the effect of consumption of a EVOO-enriched diet on amyloid- and tau-related pathological alterations that are associated with the progression of AD and cerebral amyloid angiopathy (CAA) in TgSwDI mice [170]. This model exhibit early-onset and robust Aβ pathology combined with cerebrovascular changes [171]. Three experimental groups were established: a control group fed with a standard diet, and two experimental groups fed with an EVOO-enriched diet either for 3 months beginning at an age of 4 months (EVOO-3m) or for 6 months beginning at and age of 1 month (EVOO-6m). The results showed that Aβ burden was lower in the EVOO-fed groups in comparison with control animals. Both treatments reduced the total Aβ40 and Aβ42 levels, as well as Aβ plaques observed in the hippocampus of mice. In addition, Aβ colocalization with collagen–IV (a marker of microvessels) in the hippocampus, revealed a significant reduction in the percentage of Aβ-immunoreactive microvessels in EVOO-6m mice, while in the EVOO-3m group only a trend towards a diminution was observed. The ^125^I-Aβ40 clearance in brain was improved in both EVOO-fed groups due in part to enhanced removal of Aβ across the blood-brain barrier, and P-gp and LRP-1 levels were also up-regulated. Another mechanism for Aβ clearance in the brain is the ApoE- dependent pathway [172,173] whose effectors are ABCA1 and ApoE. An increase in the levels of both proteins was observed in both treated groups. In addition, ligand-activated nuclear receptors, as PPARγ and LXRs which act inducing ABCA1 and ApoE, were studied. Levels of both proteins increased in EVOO-6m, while only PPARγ was augmented in EVOO-3m. Besides the improved clearance of Aβ, the APP process was studied by analyzing the levels of sAPPα and sAPPβ. sAPPα, a non amyloidogenic marker, was increased while sAPPβ, the amyloidogenic peptide, was diminished only in the EVOO-6m group. Finally, tau expression and phosphorylation were altered in EVOO-6m mice brains. Total tau, as well as, phosphorylated Ser214 and Thr212 isoforms, showed a significant decrease. In line with the results observed for all the biochemical parameters studied in EVOO-6m group, a significant improvement in hippocampus-dependent behavioral test was also observed. These animals burrowed more food pellets and got a high score in nest construction compared to control group and EVOO-3m. In conclusion this study confirms that a diet enriched with EVOO is able to palliate pathogenic aspects of this disease working through different molecular targets.

Using the same *in vivo* AD model, TgSwDI mice, Qosa *et al.* studied the effect of a 4 weeks treatment with oleocanthal through the AD pathological hallmarks [174]. A reduction in total Aβ levels and in Aβ plaques were noted in oleocanthal-treated mice compared to normal saline treated group (control). In addition, immunohistochemical analyses revealed a decrease in Aβ-immunoreactive microvessels among treated animals. The APP process was not altered in the presence of the treatment. The Aβ clearance across the BBB was improved with oleocanthal, and consequently, levels of P-gp and LRP-1 were significantly higher. The ApoE-dependent clearance pathway was up-regulated with the treatment, because ABCA1, ApoE and PPARγ showed an increase compared with the control group. Considering tau-pathology, oleocanthal did not modify total levels of tau or its phosphorylation state, which could be due to the short duration of the treatment. In addition, dysfunctional astrocytes have been recognized in AD patient’s brains. Aβ deposition can activate astrocytes in an abnormal manner to acquire a reactive phenotype and secrete cytokines and proinflammatory signals that are neurotoxic [175,176,177]. In the presence of oleocanthal, the levels of glial fibrillary acidic protein (GFAP), a marker of astrocyte inflammatory activation, was decreased as well as the levels of the interleukin-1β (IL-1β).

In order to evaluate if the beneficial effect of oleocanthal observed in TgSwDI mice could be also extended to humans, a series of experiments were performed in two human cell lines: hCMEC/D3, BBB endothelial cells, and SH-SY5Y-APP, neuronal cells which secrete Aβ. Using different concentrations of oleocanthal (0, 1, 5, 10 µM) during 72 h, an improvement in the basolateral transport of Aβ in SH-SY5Y-APP cells was observed, as well as, an increase in P-gp and LRP1 expression in hCMEC/D3 cells. Collectively the results of this study, both in mice and human cell lines, evidence that this polyphenol play a beneficial role in AD via targeting multiple pathological aspect of this disease. Taken together, all these studies confirm that VOO and its phenolic components are able to successfully prevent and palliate different pathogenic aspects in AD.

## 9. Effects of Olive Oil on Stem Cell Exhaustion

The decline in the regenerative potential of tissues is one of the most obvious characteristics in aging [1]. In the same way, a similar functional attrition of stem cells can be found in all adult stem cell compartments, like bone [178].

In old adults, the loss of balance between osteoblastogenesis and adipogenesis in bone marrow cell differentiation (with adopogenesis being predominant over osteoclastogenesis) linked to an increased level of osteoclastogenesis is a key mechanism in osteoporosis [179]. Osteoblasts and adipocytes derive from a common multipotential mesenchymal stem cell (MSC) progenitor. During aging, several agents including drugs, oxidative stress, hormones, metabolites, *etc.* can influence pluripotent MSC differentiation resulting in an age-related bone loss [180]. Santiago-Mora and colleagues have studied the effects of oleuropein on osteoblastogenesis and adipogenesis in MSCs from human bone marrow. Their results showed that oleuropein stimulated osteoblastogenesis enhancing some osteogenic gene expression markers as well as inducing osteoblast phenotypic characteristics. Stimulation of cellular matrix mineralization and inhibition of bone resorption were also shown. In addition, oleuropein displayed an inhibitory effect over the most important regulators of adipogenesis, as PPARγ2, and in the development and accumulation of fat in the cells [181]. Another study has tested the effect of apigenin, a flavonoid compound present in OO [182], in relation with bone loss in an *in vitro* model. This flavone could inhibit the production of osteoclastogenic cytokines in osteoblasts, while in osteoclasts it could inhibit bone resorption and induced apoptosis of mature cells. Moreover, apigenin could inhibit the differentiation of preadipocytes into adipocytes in favour of osteoblastic differentiation [183]. Taken together these results suggest that some components of OO could prevent age-related bone loss and osteoporosis in an *in vitro* system.

Liu and collaborators [184] have studied the potential beneficial effects of OO in relation with osteoporosis *in vivo*, both in rats and humans. The study included artificially ovariectomized 6-month-old female Sprague Dawley rats and ten patients aged 30–50 years who had undergone a hysterectomy and had bilateral ovarian and bilateral fallopian tube excised. Rats were fed with a diet enriched in OO during 12 weeks after surgery whereas patients were told take 50 mL of OO once every morning. Results showed that OO could prevent bone mineral density decline in rats and humans. In rats, OO could also lower the serum alkaline phosphatase (ALP) solubility which promotes mineralization of matrix proteins by hydrolysis of pyrophosphate and inorganic phosphate. These evidences point towards an important role of OO in osteoporosis prevention.

Hematopoiesis, a tightly regulated process, is also known to decline with age resulting in diminished production of adaptive immune cells (a process named immunosenescence) and increased incidence of anemia and myeloid malignances [185]. Hematopoiesis is maintained by a small pool of hematopoietic stem cells (HSCs) capable of undergoing self-renewal and generating mature progeny of all of the hematopoietic cell lineages [186]. Samet and collaborators tested oleuropein, apigenin 7-glucoside and luteolin 7-glucoside, polyphenols present in olive leafs and in OO, in CD34+ hematopoietic progenitor cells. Their results showed that these flavones displayed a protective effect by allowing HSCs to survive and give rise to different colonies. Furthermore, it was shown that olive phytochemicals modulate the fate of hematopoietic stem cells by enhancing their differentiation potential rather than their self-renewal [187].

Another studies have recognised OA as an effective molecule because it causes mesenchymal stem cells to secrete angiogenic mediators and regulates immunomodulatory functions [188]. Jun and collaborators have tried to deepen into this mechanism by studying the effect of OA in a skin wound-healing model [189]. Umbilical-cord-blood-derived MSCs (UCB-MSCs) were cultured and their motility *in vitro* investigated. Interestingly, cells treated with OA showed a promoted migration in a dose-response manner independently of cell proliferation. Furthermore, this effect was also investigated in an *in vivo* wound-healing model by injecting UCB-MSCs into mouse wounds. In accordance with the results obtained *in vitro*, the group given OA-treated UCB-MSCs showed an increased extent of wound closure with a complete re-epithelialization compared with the control group. Enhanced UCB-MSC motility was linked to ephrinB2, a protein required for vascular development with a crucial role in angiogenesis, and to F-actin, which has a vital role in cytoskeletal rearrangements. Collectively, these results support that OA is a very important promotor of tissue regeneration.

Endothelial progenitor cells (EPCs), which circulate peripherally in blood and originate from stem cells, play an important role in the re-endothelisation of damaged blood vessels and in the neovascularization of ischaemic tissues [190]. Angiotensin II, one of the pathological factors of hypertension, has the capacity to damage vascular endothelium as well as to disrupt the regenerative function of EPCs by inducing oxidative stress. This effect can accelerate the aging process in the cardiovascular system. Parzonko and collaborators have examined the potential protective effect of oleuropein and oleacein in angiotensin II-induced EPC senescence [42]. It was shown that both compounds have a protective effect on EPC exposed to angiotensin II, not only due to their intrinsic antioxidant properties, but also because of the stimulation of the transcription factor Nrf2 and the enhanced expression of the enzyme heme oxygenase-1 (HO-1). Nrf2 is responsible for intracellular antioxidant enzyme induction and protects cells against apoptosis, while HO-1, apart from transforming heme into carbon monoxide, iron and biliverdin, displays anti-inflammatory, anti-apoptotic and antioxidative properties [191,192].

This evidence, obtained both *in vivo* and *in vitro*, have shown that there is a relationship between stem cells and OO and its components, which seem to display a protective effect against some aging-related malignancies.

## 10. Alterations of Intercellular Communication Pathways by Olive Oil

Aging also involves changes at the level of cellular communication which can be endocrine, neuroendocrine or neuronal [1]. One of the most important intercellular communication events is inflammation. During this process, neutrophils and monocytes are recruited from circulation, infiltrate the tissue and undergo a maturation process with the production of inflammatory mediators like ROS, prostaglandins (PGs), cytokines and chemokines. All these factors cooperate to achieve healing and to restore homeostasis to the tissue [193]. However, inflammation leads to decreased immune responses with age causing greater susceptibility to infections and a chronic inflammation state called “inflammaging”. Various factors contribute to this state, including polymorphisms in the promoter regions of pro-inflammatory genes, chronic stimulation of immune cells with viruses, cellular senescence, obesity, changes in the gut microbiome as well as increased permeability from the intestine [194]. 

In this context, numerous *in vitro* studies have pointed out OO and its polyphenols as a source of beneficial health properties. HT exerts anti-inflammatory effects probably through the suppression of cyclooxygenase-2 (COX-2) and inducible nitric oxide synthase (iNOS) expression in human monocytic (THP-1) cells activated by lipopolysaccharides (LPS) [114]. Similar results were shown in J774 murine macrophages stimulated with LPS, where HT down-regulates iNOS and COX-2 gene expression by preventing NF-κB, STAT-1α and IRF-1 activation mediated through LPS-induced ROS generation [195]. Complementary studies, carried out in freshly isolated human monocytes activated with phorbol 12-myristate 13-acetate (PMA), show that HT is able to reduce superoxide anions as well as inhibit the accumulation of prostaglandin E2 (PGE2), an important inflammatory mediator, in the culture medium that is probably a direct consequence of the reduced expression of COX2. Moreover, HT increased production of tumor necrosis factor-alpha (TNFα) by the monocytes. These results suggest that the health effects of OO phenols may be related to their ability to modulate the production of pro-inflammatory molecules, a property common to non-steroidal anti-inflammatory drugs [193]. However, HT is not the only compound of OO that has anti-inflammatory properties. Some evidences have shown that the COX2 mRNA was also efficiently inhibited by OO secoiridoids [193]. One polyphenol of this group, oleuropein aglycone, also seems to inhibit TNFα induced matrix metalloproteinase 9 (MMP-9) in a monocyte cell line [196]. The phenolic compound oleocanthal has been found to share the same mechanistic anti-inflammatory pathway as the non-steroidal anti-inflammatory drug ibuprofen [153]. Moreover, the flavonoid apigenin regulates the expression of miR-155, suggested as an immune modulatory checkpoint and a main regulator of the key inflammatory molecule TNFα, during LPS induced inflammation in macrophages, thereby helping to restore immune balance [197].

Atherosclerosis is an inflammatory disease closely related to endothelial dysfunction. Early stages are characterized by the transport of LDL particles across the endothelium wall, which triggers several endothelium responses, such as the expression of adhesion molecules and the release of cytokines, which leads to the tethering, activation and attachment of lymphocytes and macrophages. *In vitro* studies have shown that OO phenolic compounds repress the gene expression of pro-atherogenic adhesion molecules via inactivation of NF-κB in endothelial cells [198]. Furthermore, these compounds show a powerful ROS scavenging activity in the presence of high oxidative stress, prevent oxidative damage to DNA, and enhance antioxidant activity in endothelial cells [199]. Recent studies have complemented this hypothesis by treating human umbilical vein endothelial cells (HUVEC) with human serum obtained after the intake of high-phenol and low-phenol VOO-based breakfasts. The treatment with serum obtained after the intake of a high-phenol VOO-based breakfast decreased the expression of inflammatory and oxidative stress-related genes as MCP-1, a pro-inflammatory cytokine that plays a critical role in atherosclerotic plaque formation, and reduce the oxidative stress in HUVEC. A high-phenol VOO-based breakfast seems to modulate positively the pathophysiological mechanisms that underlie the early atherosclerosis in the vascular endothelium by decreasing inflammation and improving the antioxidant profile [200].

The anti-inflammatory potential of VOO and its phenolic compounds has also been investigated *in vivo*. Santos-González and colleagues [201] studied how dietary oils affected the level of plasma proteins during aging in rats using a proteomic approach. Rats were fed lifelong with two experimental diets enriched in SO or VOO and significant changes in 16 proteins were highlighted with respect to aging or diet. These proteins played different roles in inflammation, lipid metabolism and transport, coagulation, platelet function and antioxidant protection. This study strongly supports that the intake of a diet rich in VOO has greater benefits than a diet rich in SO improving and maintaining an antioxidant status, an anti-inflammatory state and anti-atherogenic lipid profile during aging. Moreover, benefits have been demonstrated not only for OO but also for some of its minor constituents. Gong *et al.* has studied the effect of 20% HT (HT-20) on carrageenan-induced swelling and hyperalgesia of rat paw. Carrageenan-induced hindpaw inflammation is a neutrophil-mediated acute inflammatory response that produces hindpaw swelling, erythema and localized hyperthermia [202]. Interestingly, HT-20 inhibits acute inflammation and hyperalgesia (inflammatory pain) induced by carrageenan in rats. In addition, HT-20 appears to decrease IL-1 and TNFα, and no increase of the anti-inflammatory cytokine mRNA expression of IL-10 was observed [203]. Another study has reported the beneficial effect of EVOO on dextran sulfate sodium (DSS)-induced chronic colitis in mice by cytokine modulation and COX-2 and iNOS reduction via downregulation of p38 MAPK. A higher significant reduction in iNOS in HT-enriched EVOO diet compared with EVOO alone was observed as well [204]. Complementary results also suggest that the supplementation of refined OO with HT may be advantageous in rheumatoid arthritis with significant impact not only on chronic inflammation but also on acute inflammatory processes in rats [205]. Also, a celery-based apigenin-rich diet regulate the expression of miR-155 during LPS-induced inflammation *in vivo* [197]. 

Injuries also activate the local and systemic immune response with inflammatory reaction and complex interactions among cells and soluble mediators [206]. The role of n-9 MUFA present in OO in the modulation of the inflammatory and immune responses in the healing of cutaneous wounds was evaluated. Mice lesions treated with n-9 MUFA displayed a high level of IL-17 accompanied by increased collagen III expression at the final inflammatory phase of tissue repair, suggesting that the presence of this cytokine may be involved in accelerated wound healing. Elevated IL-10 and diminished COX2 gene expression were also found in n-9 MUFA treated wounds. IL-10 has been classically described as an important modulator of the inflammatory process, inhibiting several pro-inflammatory pathways [207]. Its production may balance the effects of IL-17, thus modulating the inflammatory process in n-9 MUFA treated wounds and contributing for the accelerated wound healing [188].

Some evidences showing the protective effect of OO have been also found directly in humans where cardiovascular diseases are clinical features of advanced atherosclerosis, and are often associated with aging [208]. It has been suggested that consumption of VOO during 3 weeks leads to a decrease of IL6 and C-reactive protein (CRP) higher than the decrease observed after refined OO consumption in patients with stable coronary heart disease, indicating an important role of minor antioxidant compounds of EVOO [209]. Moreover, EVOO consumption increased the anti-inflammatory effect of HDL and reduced the age-related decrease in anti-atherogenic activity [210]. Similar results also suggest a protective postprandial effect in healthy and hypertriacylcerolaemic subjects by reducing levels of adhesion molecules of the Ig superfamily [211]. Camargo *et al.* have concluded that intake of a polyphenol-rich VOO-based breakfast is able to repress *in vivo* expression of several pro-inflammatory genes, thereby switching activity of peripheral blood mononuclear cells to a less deleterious inflammatory profile [212]. Complementarily, Konstantinidou *et al.* reported that EVOO containing 328 mg/kg of phenolics in a 3-month intervention played a significant role in the downregulation of inflammatory genes implicated in atherosclerosis compared with an oil containing 55 mg/kg [213]. It was thus suggested that the benefits associated with a Mediterranean-type diet and OO polyphenol consumption on coronary heart disease risk can be mediated through changes in the expression of atherosclerosis-related genes.

## 11. Concluding Remarks and Perspectives

Cumulative evidence using cellular, animal and human models supports that dietary OO, and particularly VOO and EVOO, produce a series of healthy effects in many processes related with aging. Beneficial effects of OO consumption can be explained both on the basis of the its high MUFA content and on the healthy properties of its minor bioactive compounds. Effects of OO components on cells can be direct, for instance due to their intrinsic antioxidant properties, or indirect, mostly due to their ability to modulate gene expression. Among the minor constituents of VOO and EVOO contained in its non-saponifiable fraction, secoiridoids emerge by their capacity to modulate many pathways that are relevant for the aging process. Since this fraction is almost lost by refining procedures, EVOO should represent the best choice for healthy aging [214]. To this regards, it is very important to take into consideration that the amount of olive phenolics varies greatly, not only among different olive varieties, but also depending on the ripening phase, with an increase of some phenolics like HT and tyrosol and a decrease of others such as oleuropein aglycone with maturation [215,216,217,218]. Protection of mitochondrial and nuclear DNA against oxidative stress-induced damage can be exerted directly by the antioxidants contained in OO, with VOO polyphenols being particularly active. This action can be further increased by virtue of their ability to sustain endogenous antioxidant systems (both enzymatic and nonenzymatic) resulting in the attenuation of aging-related increase of lipid peroxidation in post-mitotic tissues, the most relevant tissues in aging, and contributing to the maintenance of genomic stability, particularly in the case of mitochondrial DNA. Although a positive relationship of leukocyte telomere length and Mediterrranean diet adherence has been also reported, studies directly evaluating the effects of OO intake on leukocyte telomere length and their effects on telomerase activity are absent, which warrants further investigation. Ethnicity may be also an important factor that must be taken into account to understand the role of Mediterrranean diet (and possibly of OO) on telomere length. OO may induce epigenetic changes both by means of its MUFA content and by its phenolic compounds. The effect of EVOO on proteostasis is apparently age and tissue-specific. Secoiridoids enhance the expression of chaperones and are important modulators of proteostasis and nutrient sensing alterations with aging. Research carried out by different groups with different models support the potential role that OO and particularly some of its phenolic compounds play in preventing and palliating some aging-associated diseases as diabetes. Of note, oleuropein produced antidiabetic effects in animal and cellular models and, interestingly, an olive leaf extract reproduced these effects in middle-aged overweight men, who are more prone to be insulin resistant. However, it is important to note that compounds contained in EVOO could target differentially mTOR signaling, one of the most important regulator of nutrient sensing which inhibition is associated with enhanced longevity. In this way, whereas both oleic acid and HT seem to activate mTOR signaling, oleocanthal inhibits this pathway which may be a major contributor to the healthy actions of this compound. Mediterranean diet is also associated with improvements of cognitive decline in aging which could be related with healthy effects of oleocanthal as recognized in mice and human cell lines. Secoiridoids as oleuropein as well as other olive phytochemicals and OA also appear to exert positive actions on stem cells function and have been recognized as very important promotors of tissue regeneration both *in vivo* and *in vitro*. Finally, inflammation is another major target of OO which can be explained by the anti-inflammatory action of HT and OO secoiridoids. Translation of these observations, particularly those involving *in vitro* approaches (which miss the effects of phenolic metabolites) and those using extracts or purified compounds at nonphysiological concentrations, to the actual effects of dietary OO on human aging should be carried out with caution. In addition, when investigations have been carried out with extracts, it is not possible to ascribe the observed effects to a particular compound [9]. In any case, the recognition that positive effects of OO are exerted on virtually all the proposed hallmarks of aging in both organisms and cellular models could help us to understand the molecular basis of health improvement, reduced risk of aging-associated diseases, and increased longevity which have been associated with consumption of a typical Mediterranean diet containing OO as the predominant fat source. Future research on this interesting topic is clearly warranted.
